# MicroRNA-19b is a potential biomarker of increased myocardial collagen cross-linking in patients with aortic stenosis and heart failure

**DOI:** 10.1038/srep40696

**Published:** 2017-01-16

**Authors:** Javier Beaumont, Begoña López, Susana Ravassa, Nerea Hermida, Gorka San José, Idoia Gallego, Félix Valencia, Juan José Gómez-Doblas, Eduardo de Teresa, Javier Díez, Arantxa González

**Affiliations:** 1 Program of Cardiovascular Diseases, Centre for Applied Medical Research, University of Navarra, 31008 Pamplona, Spain; 2 IdiSNA, Navarra Institute for Health Research, 31008 Pamplona, Spain; 3 Institute of Experimental and Clinical Research, University of Louvain (UCL) Medical School, B-1200 Brussels, Belgium; 4 Division of Cardiology, Virgen de la Victoria University Hospital, 29010 Málaga, Spain; 5 Department of Cardiology and Cardiovascular Surgery, University of Navarra Clinic, 31008 Pamplona, Spain

## Abstract

This study analyzed the potential associations of 7 myocardial fibrosis-related microRNAs with the quality of the collagen network (e.g., the degree of collagen fibril cross-linking or CCL) and the enzyme lysyl oxidase (LOX) responsible for CCL in 28 patients with severe aortic stenosis (AS) of whom 46% had a diagnosis of chronic heart failure (HF). MicroRNA expression was analyzed in myocardial and blood samples. From the studied microRNAs only miR-19b presented a direct correlation (p < 0.05) between serum and myocardium. Compared to controls both myocardial and serum miR-19b were reduced (p < 0.01) in AS patients. In addition, miR-19b was reduced in the myocardium (p < 0.01) and serum (p < 0.05) of patients with HF compared to patients without HF. Myocardial and serum miR-19b were inversely correlated (p < 0.05) with LOX, CCL and LV stiffness in AS patients. In *in vitro* studies miR-19b inhibition increased (p < 0.05) connective tissue growth factor protein and LOX protein expression in human fibroblasts. In conclusion, decreased miR-19b may be involved in myocardial LOX up-regulation and excessive CCL, and consequently increased LV stiffness in AS patients, namely in those with HF. Serum miR-19b can be a biomarker of these alterations of the myocardial collagen network in AS patients, particularly in patients with HF.

Aortic valve stenosis (AS) is the most common valve disease in western countries, and its prevalence is age-dependent affecting 3–4% of the population over 75 years old^1^. One of the main adverse consequences of AS is the development of heart failure (HF)^2^.

Myocardial fibrosis plays a crucial role in the process of myocardial remodelling in patients with AS, as well as in the development of their clinical manifestations^3^,^4^,^5^, and has a detrimental impact on the improvement in cardiac function, clinical status and long-term survival after aortic valve replacement^6^,^7^,^8^. Myocardial fibrosis is characterized not only by the increase in the quantity of collagen fibers (i.e., the fraction of myocardium occupied by collagen tissue or CVF) but also by changes in their quality (i.e., the degree of cross-linking among collagen fibrils or CCL). The degree of CCL determines the stiffness of collagen fibers and their resistance to degradation by matrix metalloproteinases^9^, and may influence cardiac function and clinical outcome. For instance, in HF patients it has been shown that an increase in CCL is associated with increased left ventricular (LV) chamber stiffness, elevated left-sided filling pressures and the development of diastolic dysfunction^10^,^11^,^12^, as well as with a higher risk for hospitalization for HF^13^. Interestingly, myocardial lysyl oxidase (LOX), the enzyme involved in fibrillary cross-linking^14^, has been reported to be associated with both CCL and left-sided filling pressures in HF patients^11^,^12^.

Several studies have demonstrated that changes in the expression of some microRNAs are associated with myocardial fibrosis (for review see ref. 15). For instance, increased expression of the pro-fibrotic microRNAs miR-21, miR-208a and miR-499-5p^16^,^17^,^18^, and decreased expression of the anti-fibrotic microRNAs miR-29, miR-19b, miR-1 and miR-133a^19^,^20^,^21^,^22^,^23^ have been reported in the myocardium in different cardiac diseases. Interestingly, the circulating levels of some of these microRNAs have been found to be altered in cardiac patients^16^,^24^,^25^. Nevertheless, the association of myocardial and circulating microRNAs with both the quantity and the quality of fibrotic tissue in cardiac patients has not been investigated.

Therefore, this study has been designed to analyze the association of the circulating and myocardial expression of the 7 previously mentioned microRNAs with myocardial CVF, CCL and LOX in patients with severe AS with and without HF. In addition, *in vitro* experiments have been performed in human adult fibroblasts with the microRNA of interest.

## Results

### Clinical characteristics

Forty-six percent of AS patients presented HF. The clinical and echocardiographic characteristics of AS patients classified according to the presence or absence of HF are shown in Tables 1 and 2, respectively. Plasma NT-proBNP was increased (p < 0.01) in AS patients with HF compared with those without HF (Table 1), even after controlling for age, sex and EF (p ≤ 0.01). The LV stiffness constant (K_LV_) was higher (p < 0.01) in HF patients compared to non-HF patients (Table 2), even after controlling for age, sex and EF (p ≤ 0.03).

### Myocardial fibrosis

As previously published, CVF was increased in AS patients compared with control subjects (18.41 ± 2.23% vs 1.95 ± 0.07%, p < 0.001), and in all patients the CVF value was above the upper limit of normality in control subjects (95% confidence interval for control subjects: 2.39%)^26^. In addition, AS patients presented a three-fold increase (p < 0.001) in soluble collagen (2.00 ± 0.18 μg/mg) and a six-fold increase (p < 0.001) in insoluble collagen (6.15 ± 0.51 μg/mg) as compared with control subjects (0.66 ± 0.17 and 0.96 ± 0.24 μg/mg, respectively). As a consequence, CCL was increased in AS patients compared with control subjects (3.79 ± 0.40 vs 1.22 ± 0.08, p < 0.001).

It has been previously reported that the expression of LOX was undetectable in myocardial samples from controls, whereas this enzyme was strongly expressed in the myocardium of AS patients^26^. Moreover, LOX expression was directly correlated with CCL (r = 0.703, p < 0.001), and CVF (r = 0.409, p < 0.05), in all AS patients (Supplementary Figure S1). Moreover, K_LV_ was directly correlated with CCL (r = 0.456, p < 0.05), and LOX (r = 0.507, p < 0.01) in all AS patients (Supplementary Figure S2).

AS patients with HF presented increased (p < 0.01) CVF, CCL and LOX compared to non-HF patients (Table 3), even after controlling for age, sex and EF (p ≤ 0.03).

### microRNA expression

From the 7 microRNAs assessed, miR-133a, miR-21 and miR-19b were detected both in myocardial and serum samples from AS patients and control subjects, whereas the expression of miR-29b, miR-1, miR-208a and miR-499-5p was under the limit of detection in serum samples from AS patients and control subjects. Therefore, we focused our study on the former 3 microRNAs.

**Figure 1:**
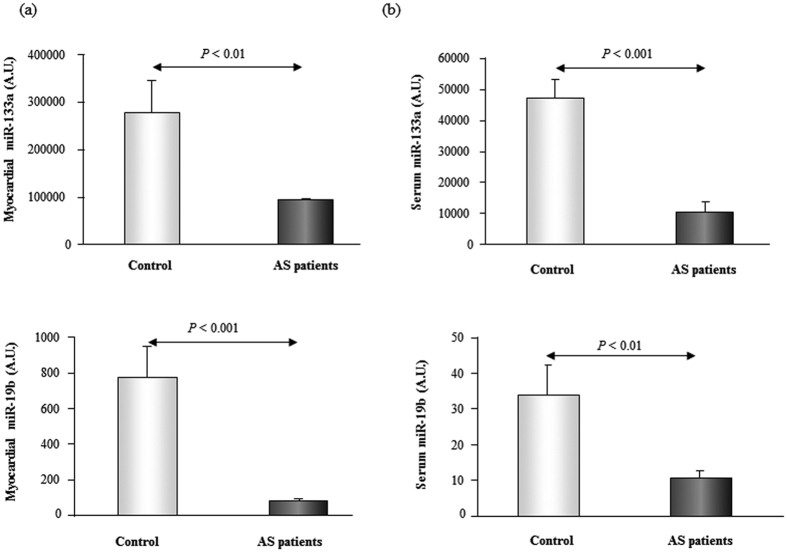
The expression of miR-133a and miR-19b is decreased in myocardium and serum from aortic stenosis patients. Relative expression of miR-133a and miR-19b in myocardium [Panel (a)] and serum [Panel (b)] from patients with aortic stenosis (AS) and control subjects. Data are shown as mean ± SEM. A.U. means arbitrary units.

The expression of miR-133a and miR-19b was reduced in the myocardial and serum samples from AS patients compared to control subjects (Fig. 1). No differences were found in serum miR-21 expression between AS patients and control subjects (data not shown), and thus no further analysis of this microRNA was performed.

No associations were found between myocardial and serum levels of miR-133a. In contrast, a direct correlation was observed for miR-19b between its expression in myocardial tissue and in peripheral blood in all AS patients (r = 0.375, p < 0.05). Thus, only the associations of this microRNA with cardiac parameters were furtherly evaluated.

### Associations of miR-19b with cardiac parameters

**Figure 2:**
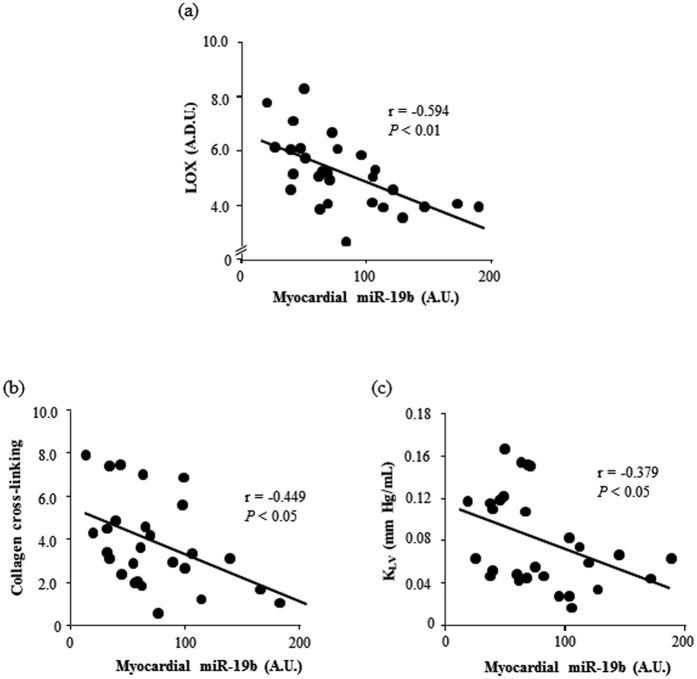
Myocardial miR-19b is inversely correlated with LOX, collagen cross-linking and left ventricular stiffness in aortic stenosis patients. Inverse correlations between myocardial miR-19b expression and myocardial LOX protein [linear fit: −1.823x + 6.761; panel (a)], collagen cross-linking [linear fit: −2.224x + 5.626; panel (b)] and left ventricular chamber stiffness constant (K_LV_) [linear fit: −0.042x + 0.113; panel (c)] in patients with aortic stenosis. A.D.U. means arbitrary densitometric units, A.U., arbitrary units. *P* values are for bivariate correlation analysis.

**Figure 3:**
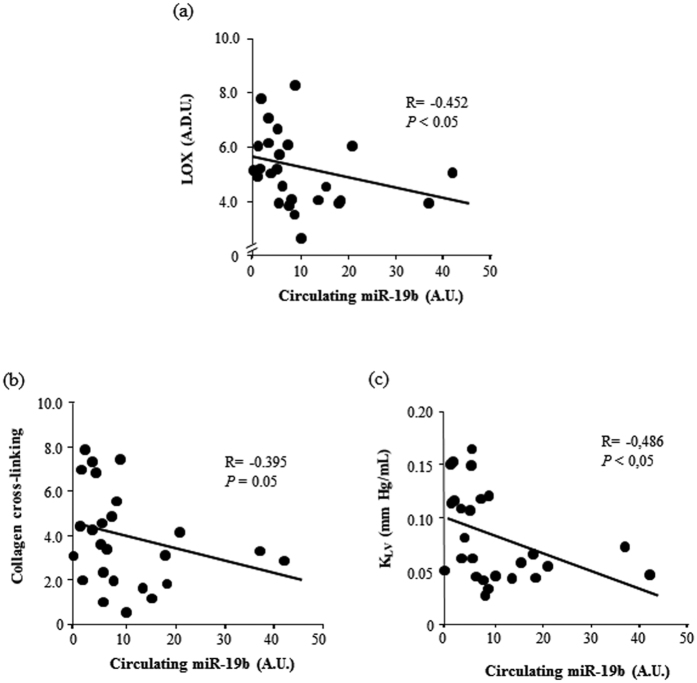
Serum miR-19b is inversely correlated with LOX, collagen cross-linking and left ventricular stiffness in aortic stenosis patients. Inverse correlations between serum miR-19b expression and myocardial LOX protein [linear fit: −0.037x + 5.587; panel (a)], collagen cross-linking [linear fit: −0.059x + 4.505; panel (b)] and left ventricular chamber stiffness constant (K_LV_) [linear fit: −0.002x + 0.100; panel (c)] in patients with aortic stenosis. A.D.U. means arbitrary densitometric units, A.U., arbitrary units. *P* values are for bivariate correlation analysis.

Myocardial miR-19b was inversely correlated with CVF (r = −0.409, p < 0.05), as well as with LOX, and CCL in AS patients (Fig. 2). Moreover, myocardial miR-19b was inversely correlated with K_LV_ in AS patients (Fig. 2). Serum levels of miR-19b were also inversely correlated with LOX, CCL and K_LV_ in AS patients (Fig. 3).

Interestingly, AS patients with HF showed decreased myocardial (p < 0.01) and serum (p < 0.05) miR-19b, compared to AS patients without HF (Table 3). Moreover, logistic regression analysis showed that decreased myocardial miR-19b was associated with the presence of HF (OR 0.949, 95% CI 0.91–0.99; p < 0.05), independently of age, gender and ejection fraction.

### *In vitro* studies

As expected, miR-19b expression was decreased (p < 0.01) in HDF cells transfected with antimiR-19b (0.01 ± 0.01 A.U.) compared to those transfected with control oligonucleotides (1.06 ± 0.18 A.U.).

**Figure 4:**
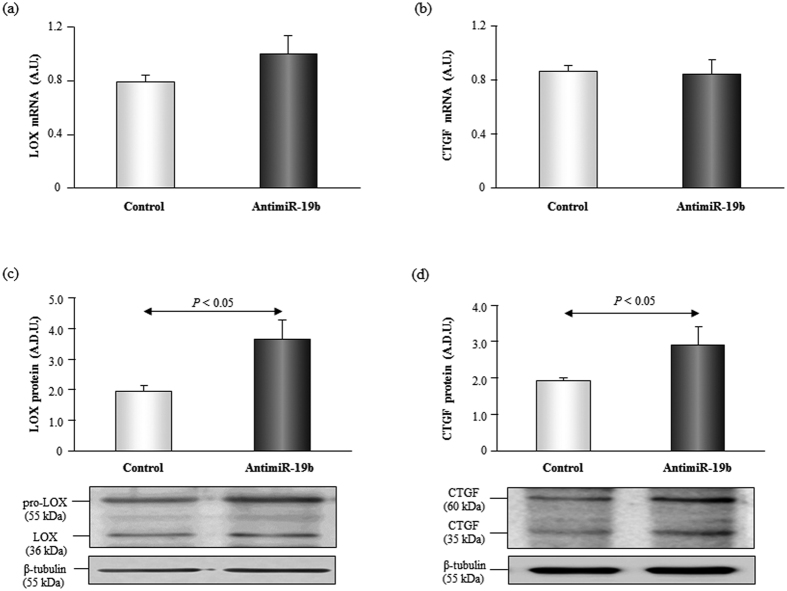
The inhibition of miR-19b induces an increase in CTGF and LOX protein in human dermal fibroblasts. Expression of LOX mRNA and protein [panels (a) and (c)] and CTGF mRNA and protein [panels (c) and (d)] in adult human dermal fibroblasts transfected with control oligonucleotides or with anti-miR-19b. Representative images of Western blots for pro-LOX (55 kDa) and LOX (36 kDa), CTGF (60 kDa and 36 kDa) and β-tubulin (55 kDa) in both groups of transfected cells. Data are shown as mean ± SEM. A.D.U. means arbitrary densitometric units.

Some studies have shown that active LOX is also present in the cytosol of fibroblasts^27^, in fact in cell lysates we mainly detected the pro-enzyme but we could also find the active form. Inhibition of miR-19b significantly increased (p < 0.05) the expression of LOX protein in HDF cells (Fig. 4). Although a slight increase in LOX mRNA levels was observed in HDF cells transfected with antimiR-19b, the differences were not statistically significant (Fig. 4).

In silico analysis according to the databases mentioned in the Methods section indicated that CTGF is a potential target for miR-19b, therefore, we analyzed its protein expression in HDF transfected cells. CTGF protein expression was increased (p < 0.05) in HDF cells transfected with antimiR-19b compared to control cells, whereas no changes were found in CTGF mRNA levels (Fig. 4).

## Discussion

The main findings of the present study are the following: (1) Decreased myocardial and serum miR-19b expression is present in AS patients, namely in those with HF; (2) Both myocardial and serum miR-19b are inversely associated with myocardial LOX protein and CCL, as well as with LV stiffness in AS patients; (3) miR-19b inhibition increased the expression of CTGF and LOX protein in human adult fibroblasts.

Although down-regulation of myocardial miR-19b expression has been reported previously in AS patients^23^, findings here reported expand this information on this microRNA showing that miR-19b was abnormally decreased not only in the myocardium but also in the blood of these patients, and namely in those with HF. In addition, we report for the first time that decreased miR-19b was associated with increased myocardial LOX and CCL in AS patients. These associations suggest that miR-19b may modulate the enzyme LOX which determines CCL^14^. In fact, our *in vitro* data suggest a role of miR-19b in the regulation of LOX expression in human fibroblasts. Although the underlying mechanisms need to be fully elucidated, we report that miR-19b inhibition also increases CTGF expression in fibroblasts. Coherently, miR-19b has been shown to regulate CTGF in ageing-associated HF both in a rodent model and in patients^20^. Interestingly, it has been reported that CTGF regulates LOX protein expression and activity in cardiac fibroblasts^28^,^29^. Therefore, it may be speculated that miR-19b might control LOX expression in human fibroblasts via CTGF regulation.

MiR-19b belongs to the miR-17-92 cluster which includes miR-17, miR-18, miR-19a, miR-19b, miR-20 and miR-92^30^. Different components of the 17–92 cluster have been reported to regulate angiogenesis^31^,^32^, cardiomyocyte proliferation^33^ and myocardial fibrosis^20^. A recent study has shown that a decreased expression of all members of the cluster, is involved in the development of pulmonary fibrosis^34^. Three paralog miR-17-92 clusters have been described in humans, in chromosomes 13, X and 7^35^. Although some members of the cluster are located in chromosome X (e.g. miR-19b-2) no differences were observed between males and females in miR-19b expression neither in myocardium nor in serum (Supplementary results).

The potential pathophysiological impact of the association between miR-19b, LOX and CCL is given by our observation that these parameters were associated with LV chamber stiffness and that all of them were associated with the presence of HF in AS patients. In this regard, CCL has been found to be directly associated with cardiac dysfunction and LV chamber stiffness in hypertensive patients with HF^12^. Moreover, CCL was also associated with some hallmarks of the severity of HF (i.e. estimated pulmonary capillary wedge pressure and NT-proBNP) and with a higher risk of hospitalization for HF in patients with this syndrome^12^,^13^. Taken together, all these data support the hypothesis that myocardial miR-19b could be involved in LV dysfunction by facilitating LOX-mediated CCL that results in the formation of stiff collagen fibers in the myocardium of patients with AS and HF. This is further supported by the association between myocardial miR-19b and the presence of HF observed in AS patients, independently of possible confounding factors like age, sex and ejection fraction.

On the other hand, the associations here reported between circulating miR-19b and myocardial parameters, namely CCL, may be of potential clinical interest. In fact, whereas several circulating molecules have been proposed as biomarkers of the quantity of myocardial fibrosis (i.e. CVF)^36^, up to now there is only one proposed biomarker of CCL (i.e. the serum ratio of C-terminal telopeptide of collagen type I to matrix metalloproteinase-1)^13^. Therefore, although validation studies in larger independent cohorts are necessary, circulating miR-19b could be another biomarker of the quality of myocardial fibrosis (i.e. CCL), at least in AS patients. In this regard, it must be noted that a direct correlation was found between myocardial and serum miR-19b suggesting the partial cardiac origin of serum miR-19b in AS patients.

Regarding the other microRNAs found altered in this study, we confirmed that miR-133a is down-regulated in the myocardium of patients with AS^37^,^38^. However, the lack of association of this microRNA between the myocardium and the serum does not support its potential role as biomarker of myocardial fibrosis in AS patients. Finally, although Villar *et al*.^16^ showed that circulating and myocardial miR-21 levels were increased in AS patients no changes in circulating miR-21 were observed in the current study, probably due to differences in the clinical characteristics of the patients (e.g. differences in the frequency of patients treated with β-blockers) that might influence the expression of this microRNA^39^.

### Study limitations

We are aware that this study presents some limitations. First, it involved a relatively low number of patients, but since it was a pilot study involving myocardial biopsies, the design is appropriate. Second, we performed biopsies of the interventricular septum to assess the effects of LV pressure overload. However, as previously shown^40^, fibrosis present in the septum from pressure overloaded human hearts is representative of fibrosis existing in the free wall. Third, although the methodology was standardized, differences in geographical sites between sample collection and processing could contribute to variability in the results. Moreover, the direct comparison of microRNA expression in myocardium and serum could be affected by the use of different types of controls for microRNA normalization: biological [U6] for tissue and technical [cel-miR-39] for the serum. However, these methods for normalization are commonly used^16^,^26^,^41^. Fourth, we have focused our study in some of the main microRNAs involved in myocardial fibrosis, due to sample limitation, nevertheless we cannot discard that other fibrosis-related microRNAs could play a role in this process^37^,^42^,^43^. Fifth, although previously used^16^, the isolation of serum microRNAs using Trizol is not the optimal method and several small RNAs may have been lost during the extraction procedure.

## Conclusions

We report that myocardial levels of miR-19b were abnormally decreased in AS patients and were associated with an excess of LOX and CCL, as well as increased LV stiffness, namely in those patients with HF. Although descriptive in nature these findings raise the possibility that miR-19b participates in the alterations of the myocardial collagen that impair LV mechanics and contribute to HF in AS.

On the other hand serum miR-19b was also decreased in AS patients and was associated with its myocardial expression. Moreover, decreased serum miR-19b was also associated with excessive myocardial LOX and CCL, and increased LV stiffness, especially in patients with HF. Thus, circulating miR-19b emerges as a potential biomarker of the alterations of myocardial collagen present in AS patients with HF.

## Methods

### Patients and sample acquisition

All subjects gave written informed consent to participate in the study, and the Institutional Ethics Committee of the Virgen de la Victoria University Hospital (Málaga, Spain) approved the protocol. The study conformed to the principles of the Declaration of Helsinki.

The study population consisted of 28 patients with severe isolated AS referred for surgical aortic valve replacement to the Virgen de la Victoria University Hospital as previously described^26^. The diagnosis of HF was made in 13 patients (46%) on a clinical basis, by the presence of, at least, 1 major and 2 minor Framingham criteria^44^ and was confirmed by echocardiographic alterations in cardiac morphology and function and by the presence of elevated levels of the amino-terminal pro-brain natriuretic peptide (NT-proBNP)^45^. Patients with cardiac valve diseases other than AS, history of acute myocardial infarction, a significant stenosis (>50%) in 1 or more coronary arteries in the angiography, or disorders associated with alterations in collagen turnover were excluded after complete medical examination. (For details see the Supplementary Data).

Blood and myocardial samples were obtained during surgical replacement of the aortic valve. Blood samples were obtained from the left antecubital vein at the time of valve replacement and stored at −20 °C. Two myocardial samples were obtained from the interventricular septum, one sample was immediately fixed in 4% buffered formalin and embedded in paraffin for the determination of histomorphological parameters, and the other sample was divided into two smaller pieces and frozen separately in liquid nitrogen following different processing for protein or RNA isolation.

Nineteen healthy subjects were used as controls for venous blood determinations, and ten necropsies from subjects who died from causes other than cardiovascular diseases were used as controls for myocardial parameters. All of these subjects were collected at the University of Navarra Clinic.

### Echocardiographic assessment

Parameters assessing LV morphology and function were measured from M-mode recordings using leading edge methodology according to the American and European Societies of Echocardiography criteria. (For details see the Supplementary Data).

### NT-proBNP determination

Plasma NT-proBNP was measured by an enzyme-linked immunosorbent assay using a commercial kit (Biomedica Gruppe). All determinations were performed in duplicate. The inter- and intra-assay coefficients of variation were 8 and 5%, respectively.

### Myocardial fibrosis assessment

CVF was determined by quantitative morphometry with an automated image analysis system (Cell, Soft Imaging System) in sections stained with Picrosirius Red (Sirius Red F3BA in aqueous picric acid), as previously reported^40^. All measurements were performed in duplicate by 2 independent observers. The inter- and intra-observer coefficients of variation were <4%.

To assess CCL an enzymatic and colorimetric procedure was used to evaluate insoluble (cross-linked) and soluble collagen, as previously described^11^. The CCL was calculated as the ratio between insoluble and soluble collagen. (For details see the Supplementary Data).

Protein expression of LOX was analyzed by Western blot in myocardial samples visualized with a chemiluminescence system (GE Healthcare) and analyzed using an automatic densitometer (GS-800 Calibrated Densitometer, Bio-Rad). (For details see the Supplementary Data).

### Myocardial microRNA analysis

Myocardial RNA was isolated with TRIzol reagent (Invitrogen) according to manufacturer’s recommendations (using 10 μg of glycogen to enhance the efficiency of RNA extraction) and the pellet was resuspended in 40 μL of nuclease-free water. RNA concentration was determined with a NanoDrop ND-1000 Spectrophotometer. Reverse transcription (RT) of myocardial microRNAs was performed with a fixed volume of 4 μL of RNA using the TaqMan MicroRNA Reverse Transcription Kit (Applied Biosystems) and a pool of specific primers for miR-19b, miR-133a, miR-21, miR-29, miR-1, miR-208a, miR-499-5p and for snU6 (each primer diluted 1:100 in the pool). The cDNA obtained was pre-amplified for 12 cycles using the TaqMan PreAmp Master Mix Kit (Applied Biosystems) and a pool of specific TaqMan microRNA assays (each assay diluted 1:100 in the pool) as previously described^26^. PCR of these pre-amplified microRNAs was performed with specific TaqMan microRNA assays. The fluorescent signal was detected with a 7900HT Fast Real-Time PCR System (Applied Biosystems) and analyzed with the SDS 2.2.2 software. The snU6 RNA was analyzed with a specific TaqMan probe as an endogenous control. The fluorescent signal was detected and analyzed as previously described. MicroRNA expression was calculated by the 2^−ΔΔCt^ method relative to snU6 RNA and data were presented as arbitrary units (A.U.).

### Serum microRNA analysis

Circulating microRNA levels were determined by real-time RT-PCR. RNA was isolated from 150 μL of serum with TRIzol LS reagent (Invitrogen) according to manufacturer’s recommendations (using 10 μg of glycogen to enhance the efficiency of RNA extraction). Synthetic versions of the *C. elegans* microRNA 39 (cel-miR-39) were spiked into serum, at a constant amount of 25 fmol, after the addition of the Trizol LS reagent to the samples. The RNA pellet was resuspended in 40 μl of nuclease-free water and RNA concentration was determined with a NanoDrop ND-1000 Spectrophotometer (NanoDrop Tech). RT was performed with a fixed volume of 4 μL of RNA using the TaqMan MicroRNA Reverse Transcription Kit (Applied Biosystems) as indicated for myocardial tissue. The cDNA obtained was pre-amplified as described above for myocardial tissue and amplified using the specific TaqMan microRNA assays (Life Technologies). The fluorescent signal was detected and analyzed as previously described. The Ct values from real-time polymerase chain reaction (PCR) assays greater than 40 were treated as 40. MicroRNA expression was calculated by the 2^−ΔΔCt^ method relative to cel-miR-39 expression and presented as arbitrary units (A.U.).

### In silico analysis

Prediction of potential targets of the miRNA of interest was performed using the databases microRNA.org (http://www.microrna.org), miRDB (http://mirdb.org/miRDB), Targetscan 6.2 (http://www.targetscan.org), mirTarBase (http://mirtarbase.mbc.nctu.edu.tw) and TarBase (http://diana.imis.athena-innovation.gr).

### *In vitro* studies

Adult human dermal fibroblasts (HDF) (Thermo Fisher Scientific) were transfected with antimiR-19b or negative control oligonucleotides (16 nM) using RNAiMax Lipofectamine (Thermo Fisher Scientific) according to manufacturer’s recommendations, and total protein and RNA were isolated after 24 hours. Intracellular protein was isolated with the M-PER mammalian protein extraction reagent (Thermo Fisher Scientific) whereas the RNA was isolated with the Maxwell® 16 LEV simply RNA Purification Kit (Promega). The mRNA expression of connective tissue growth factor (CTGF) and LOX was analyzed by RT-PCR using specific Taqman assays (Thermo Fisher Scientific and Integrated DNA Technologies). Protein expression of CTGF and LOX was analyzed by Western blot. (For details see the Supplementary Data).

### Statistical analysis

To analyze the differences between controls and AS patients, between patients with and without HF, and between two groups of cells, a Student’s t test for unpaired data was used once normality was demonstrated (Shapiro-Wilks test); otherwise, a non-parametric test (Mann-Whitney *U* test) was used. The association between continuously distributed variables was tested by bivariate correlation analysis. Non-parametric distributed variables were examined after logarithmic transformation. Logistic regression analysis was performed to evaluate the association between quantitative and qualitative variables and the potential influence of age, gender and the ejection fraction was considered in the analysis. Data are expressed as mean ± standard error of the mean (S.E.M.), and percentage of patients. All tests were two sided, and a value of p < 0.05 was considered statistically significant. The analysis was performed using the SPSS 15.0 statistical package (SPSS Inc.).

## Additional Information

**How to cite this article**: Beaumont, J. *et al*. MicroRNA-19b is a potential biomarker of increased myocardial collagen cross-linking in patients with aortic stenosis and heart failure. *Sci. Rep.* **7**, 40696; doi: 10.1038/srep40696 (2017).

**Publisher's note:** Springer Nature remains neutral with regard to jurisdictional claims in published maps and institutional affiliations.

## Supplementary Material

Supplementary Dataset

## Figures and Tables

**Table 1:** Clinical characteristics in aortic stenosis patients classified according to the presence or absence of heart failure.

Parameters	Non-HF (n = 15)	HF (n = 13)
Age, years	71 ± 2	72 ± 2
Sex (male/female), N	3/12	5/8
***Co-morbidities***		
Left ventricular hypertrophy, n (%)	9 (60)	8 (62)
Diabetes mellitus, n (%)	6 (40)	4 (31)
Atrial fibrillation, n (%)	3 (20)	4 (31)
Coronary heart disease, n (%)	6 (40)	3 (23)
***Clinical manifestations***		
Angina, n (%)	11 (73)	3 (23)*
Syncope, n (%)	2 (13)	1 (8)
Dyspnea, n (%)	15 (100)	13 (100)
***Treatment***		
Diuretics, n (%)	9 (60)	12 (92)^†^
β-blockers, n (%)	7 (46)	4 (31)
ACEI or ARA, n (%)	2 (13)	6 (46)
Anticoagulants or antiaggregants, n (%)	10 (67)	10 (77)
***Biochemical parameters***		
NT-proBNP, pmol/L	628.59 ± 82.48	1120.67 ± 122.641*

HF means heart failure; N, number of patients; β-blockers, beta-blockers; ACEI, angiotensin converting enzyme inhibitors; ARA, angiotensin II type 1 receptor antagonists; NT-proBNP, amino-terminal pro-brain natriuretic peptide. Values are expressed as mean ± SEM, and number or percentage of patients. *P < 0.01 vs Non-HF patients, ^†^*P* < 0.05 vs Non-HF patients.

**Table 2:** Echocardiographic characteristics in aortic stenosis patients classified according to the presence or absence of heart failure.

Parameters	Non-HF patients (n = 15)	HF patients (n = 13)
AVA, cm^2^	0.64 ± 0.06	0.59 ± 0.06
TPG mean, mm Hg	55.43 ± 4.52	52.01 ± 5.16
TPG max, mm Hg	82.90 ± 6.47	80.22 ± 7.69
LVMI, g/m^2^	130.87 ± 8.78	156.41 ± 13.42
RWT	0.59 ± 0.03	0.66 ± 0.05
LVESVI, mL/m^2^	25.84 ± 3.87	47.14 ± 13.73
LVEDVI, mL/m^2^	87.17 ± 7.33	99.35 ± 13.63
cESS, kdynes/cm^2^	122.70 ± 9.17	150.90 ± 24.17
mESS, kdynes/cm^2^	54.06 ± 4.65	71.47 ± 14.63
V_E_/V_A_	0.722 ± 0.074	1.470 ± 0.462
DT, ms	308.60 ± 18.37	257.77 ± 29.26*
K_LV_, mmHg/mL	0.054 ± 0.006	0.098 ± 0.013^†^
IVRT, ms	98.22 ± 10.47	100.10 ± 22.46
LVEF, %	71.30 ± 1.73	60.73 ± 6.17

HF means heart failure; AVA, aortic valve area; TPG, transvalvular pressure gradient; max, maximal; LVMI, left ventricular mass index; RWT, relative wall thickness; LVESVI, LV end-systolic volume index; LVEDVI, LV end-diastolic volume index; cESS, circumferencial end-systolic wall stress; mESS, meridional end-systolic wall stress; V_E_, maximum early transmitral velocity in diastole; V_A_, maximum late transmitral velocity in diastole; DT, deceleration time; K_LV_, LV chamber stiffness constant; IVRT, isovolumetric relaxation time; LVEF, LV ejection fraction; NT-proBNP, amino-terminal pro-brain natriuretic peptide. Values are expressed as mean ± SEM. *P < 0.05 vs Non-HF patients, ^†^*P* < 0.01 vs Non-HF patients.

**Table 3:** MicroRNA-19b expression and parameters related to myocardial fibrosis in aortic stenosis patients classified according to the presence or absence of heart failure.

	Non-HF patients (n = 15)	HF patients (n = 13)
Myocardial microRNA-19b (A.U.)	107.75 ± 10.88	57.28 ± 6.47*
Serum microRNA-19b (A.U.)	9.58 ± 1.68	5.04 ± 0.86^†^
LOX (A.D.U.)	4.39 ± 0.21	6.19 ± 0.30*
CCL	2.78 ± 0.47	4.89 ± 0.54*
CVF (%)	14.28 ± 2.76	23.17 ± 3.21*

HF means heart failure; A.U., arbitrary units; A.D.U., arbitrary densitometric units; LOX, lysyl oxidase; CCL, collagen cross-linking; CVF, collagen volume fraction. Values are expressed as mean ± SEM. *P < 0.01 vs non-HF AS patients, ^†^*P *< 0.05 vs non-HF AS patients.
