# Author Correction: MicroRNA-19b is a potential biomarker of increased myocardial collagen cross-linking in patients with aortic stenosis and heart failure

**DOI:** 10.1038/s41598-020-63179-1

**Published:** 2020-04-16

**Authors:** Javier Beaumont, Begoña López, Susana Ravassa, Nerea Hermida, Gorka San José, Idoia Gallego, Félix Valencia, Juan José Gómez-Doblas, Eduardo de Teresa, Javier Díez, Arantxa González

**Affiliations:** 1 Program of Cardiovascular Diseases, Centre for Applied Medical Research, University of Navarra, 31008 Pamplona, Spain; 2 IdiSNA, Navarra Institute for Health Research, 31008 Pamplona, Spain; 3 Institute of Experimental and Clinical Research, University of Louvain (UCL) Medical School, B-1200 Brussels, Belgium; 4 Division of Cardiology, Virgen de la Victoria University Hospital, 29010 Málaga, Spain; 5 Department of Cardiology and Cardiovascular Surgery, University of Navarra Clinic, 31008 Pamplona, Spain

Correction to: *Scientific Reports* 10.1038/srep40696, published online 16 January 2017

In the original version of this Article, the author Gorka San José was incorrectly indexed. This error has now been corrected.

