# Supplementary material for: MicroRNA-19b is a potential biomarker of increased myocardial collagen cross-linking in patients with aortic stenosis and heart failure
Source: Sci Rep. 2017 Jan 16;7:40696. doi: 10.1038/srep40696 (PMC5238428; doi:10.1038/srep40696)
Supplement: Supplementary Dataset [file srep40696-s1.doc]

**Supplementary Information**

**MicroRNA-19b is a potential biomarker of increased myocardial collagen cross-linking in patients with aortic stenosis and heart failure**

Javier Beaumont, Begoña López, Susana Ravassa, Nerea Hermida, Gorka San José, Idoia Gallego, Félix Valencia, Juan José Gómez-Doblas, Eduardo de Teresa, Javier Díez, Arantxa González.

**Supplementary results**

**microRNA expression**

When miR-19b was analyzed in serum and myocardium from AS patients classified by gender, no differences were observed between males and females neither in the myocardium (males: 72.79±12.68 A.U.; females: 88.92±10.09 A.U.) nor in serum (males: 12.10±4.29 A.U.; females: 10.03±2.35 A.U.).

**Supplementary methods**

**Patients and sample acquisition**

Severe aortic stenosis (AS) was defined in accordance with the following criteria^1^: Mean transvalvular pressure gradient (TPG mean) > 40 mm Hg, aortic valve area (AVA) < 1 cm^2^, or AVA index < 0.6 cm^2^/m^2^. Aortic valve replacement was performed due to the presence of characteristic manifestations of AS (i.e. angina, syncope, dyspnea or heart failure [HF]).

**Echocardiographic assessment**

LV mass was measured from M-mode recordings using leading edge methodology according to the American and European Societies of Echocardiography criteria^2^. LV mass index (LVMI) was calculated by dividing LV mass by body surface area. The presence of LV hypertrophy (LVH) was established when LVMI was > 149 g/m^2^ in men and > 122 g/m^2^ in women^2^. LV end-systolic and end-diastolic volume indexes (LVESVI and LVEDVI, respectively), corresponding to the LV volumes corrected by body surface area, and the LV ejection fraction (LVEF), which was calculated according to Quinones et al.^3^, were determined in all patients.

The following pulsed Doppler measurements were obtained: maximum early (V_E_) transmitral velocity in diastole, maximum late (V_A_) transmitral velocity in diastole, the deceleration time of the early mitral filling wave (DT), and the isovolumetric relaxation time (IVRT). LV chamber stiffness constant (K_LV_) was calculated as the ratio squared of DT according to the following equation^4^: K_LV_ = (0.07 : DT)^2^. Meridional and circumferential end-systolic stress (mESS and cESS, respectively) were calculated according to the method validated by Reichek et al.^5^ as indexes of ventricular afterload.

TPG mean and maximal TPG (TPG max) were assessed in all patients using the simplified equation of Bernouilli: TPG = 4v^2^, where v represents flow velocity. AVA was assessed in all patients according to Skjaerpe et al^6^.

**Myocardial fibrosis assessment**

An enzymatic and colorimetric procedure was used to evaluate insoluble (cross-linked) collagen, as previously described^7^. First, a fast green/Sirius red assay was performed to identify and quantify total collagen. In a second step, a sircol-based assay was performed to obtain and quantify soluble collagen. The amount of insoluble collagen was calculated by subtracting the amount of soluble collagen from the amount of total collagen. Collagen cross-linking was assessed as the ratio between insoluble and soluble collagen. All measurements were performed in duplicate. The inter- and intra-assay coefficients of variation were 5 and 3%, respectively.

Myocardial protein was isolated using the M-PER mammalian protein extraction reagent (Thermo Fisher Scientific) according to the manufacturer’s recommendations, and protein expression of LOX was analyzed by Western blot. A specific rabbit polyclonal antibody against LOX (Abcam) was incubated at a dilution of 1:500. Bands were detected using peroxidase-conjugated secondary antibody (GE Healthcare). Protein expression was visualized with a chemiluminescence system (GE Healthcare) and autoradiograms were analyzed using an automatic densitometer (GS-800 Calibrated Densitometer, Bio-Rad). The blots were incubated with a monoclonal anti-β-actin antibody (Sigma) followed by as a control for loading. Data are expressed as arbitrary densitometric units (A.D.U.) relative to β-actin expression.

**In vitro studies**

Adult human dermal fibroblasts (HDF) (Thermo Fisher Scientific) were grown in DMEM low glucose medium (Thermo Fisher Scientific), and they were transfected with antimiR-19b or negative control oligonucleotides (16nM) using RNAiMax Lipofectamine (Thermo Fisher Scientific) and serum free medium Optimem (Thermo Fisher Scientific). After 6 hours the medium was replaced by fresh DMEM low glucose medium, to minimize the toxicity of lipofectamine, and HDF cells were incubated for 24 hours. After 24 hours HDF cells were divided in two parts for mRNA or protein extraction. The mRNA was isolated from the HDF cells with the [Maxwell® 16 LEV simplyRNA Purification Kit](https://www.google.es/url?sa=t&rct=j&q=&esrc=s&source=web&cd=2&cad=rja&uact=8&ved=0ahUKEwiV5qSV1fLMAhWG7hoKHWiOBUcQFghAMAE&url=https%3A%2F%2Fwww.promega.com%2Fproducts%2Frna-purification-quantitation%2Fautomated-rna-extraction-systems%2Fmaxwell-16-system-rna-purification-kits%2Fmaxwell-16-lev-simplyrna-purification-kits%2F&usg=AFQjCNGoNBDqom46lZ_ilHfsRH0l75xpRw&sig2=5lUnG2X8L2LXgxnSQe2EhQ) (Promega). The expression of miR-19b was analyzed in HDF by RT, pre-amplification and PCR as mentioned above, and those experiments in which anti miR-19b transfection induced a significant decrease (P<0.05) in miR-19b compared to control oligonucleotides were considered for further analysis. To analyze mRNA expression of connective tissue growth factor (CTGF) and LOX in HDF cells, RT was performed using the High-Capacity cDNA Reverse Transcription Kit (Thermo Fisher Scientific) and PCR was performed with a 7900 HT Fast Real-time PCR system using specific TaqMan assays for CTGF and LOX (Hs00170014 and Hs00942480_m1, respectively, Thermo Fisher Scientific), and specific assay for 18S ribosomal RNA (Hs.PT.39a22214856.g, Integrated DNA Technologies) as an endogenous control. Data were analyzed as A.U. relative to 18S ribosomal RNA.

Intracellular protein was isolated from the HDF cells with the M-PER mammalian protein extraction reagent (Thermo Fisher Scientific) and protein expression of CTGF and LOX was analyzed by Western blot in HDF cells. Specific rabbit antibodies against LOX (Abcam) and CTGF (Thermo Fisher Scientific) were incubated at a dilution of 1:500 and 1:2500 respectively, and bands were detected using a peroxidase-conjugated secondary antibody (GE Healthcare) and visualized with a chemiluminescence system (GE Healthcare). The blots were incubated with a monoclonal anti-β-tubulin antibody (Sigma) as a control for loading. Data are expressed as arbitrary densitometric units (A.D.U.) relative to β-tubulin expression.

**References**

1. Bonow, R.O., *et al.* 2008 Focused update incorporated into the ACC/AHA 2006 guidelines for the management of patients with valvular heart disease: a report of the American College of Cardiology/American Heart Association Task Force on Practice Guidelines (Writing Committee to Revise the 1998 Guidelines for the Management of Patients With Valvular Heart Disease): endorsed by the Society of Cardiovascular Anesthesiologists, Society for Cardiovascular Angiography and Interventions, and Society of Thoracic Surgeons. *Circulation.* **118,** e523-e561 (2008).

2. Lang, R.M., *et al.* Recommendations for chamber quantification. *Eur J Echocardiogr.* **7,** 79-108 (2006).

3. Quinones, M.A., Pickering, E., Alexander, J.K. Percentage of shortening of the echocardiographic left ventricular dimension. Its use in determining ejection fraction and stroke volume. *Chest.* **74,** 59-65 (1978).

4. [Garcia, M.J](http://www.ncbi.nlm.nih.gov/pubmed?term=Garcia MJ%5BAuthor%5D&cauthor=true&cauthor_uid=11158951)., *et al.* Estimation of left ventricular operating stiffness from Doppler early filling deceleration time in humans. [*Am J Physiol Heart Circ Physiol*](http://www.ncbi.nlm.nih.gov/pubmed/?term=Garcia+MJ%2C+Firstenberg+MS%2C+Greenberg+NL%2C+Smedira+N%2C+Rodriguez+L%2C+Prior+D%2C+Thomas+JD.+Estimation+of+left+ventricular+operating+stiffness+from+Doppler+early+filling+deceleration+time+in+humans.+Am+J+Physiol+Heart+Circ+Physiol.+2001%3B280%3AH554-61.)*.* **280,** H554-H561 (2001).

5. Reichek, N., *et al.* Noninvasive determination of left ventricular end-systolic stress: validation of the method and initial application. *Circulation.* **65,** 99-108 (1982).

6. Skjaerpe, T., Hegrenaes, L., Hatle, L. Noninvasive estimation of valve area in patients with aortic stenosis by Doppler ultrasound and two-dimensional echocardiography. *Circulation.* **72,** 810-818 (1985).

7. López, B., *et al.* Impact of treatment on myocardial lysyl oxidase expression and collagen cross-linking in patients with heart failure. *Hypertension.* **53,** 236-242 (2009).

**Supplementary figures**

**
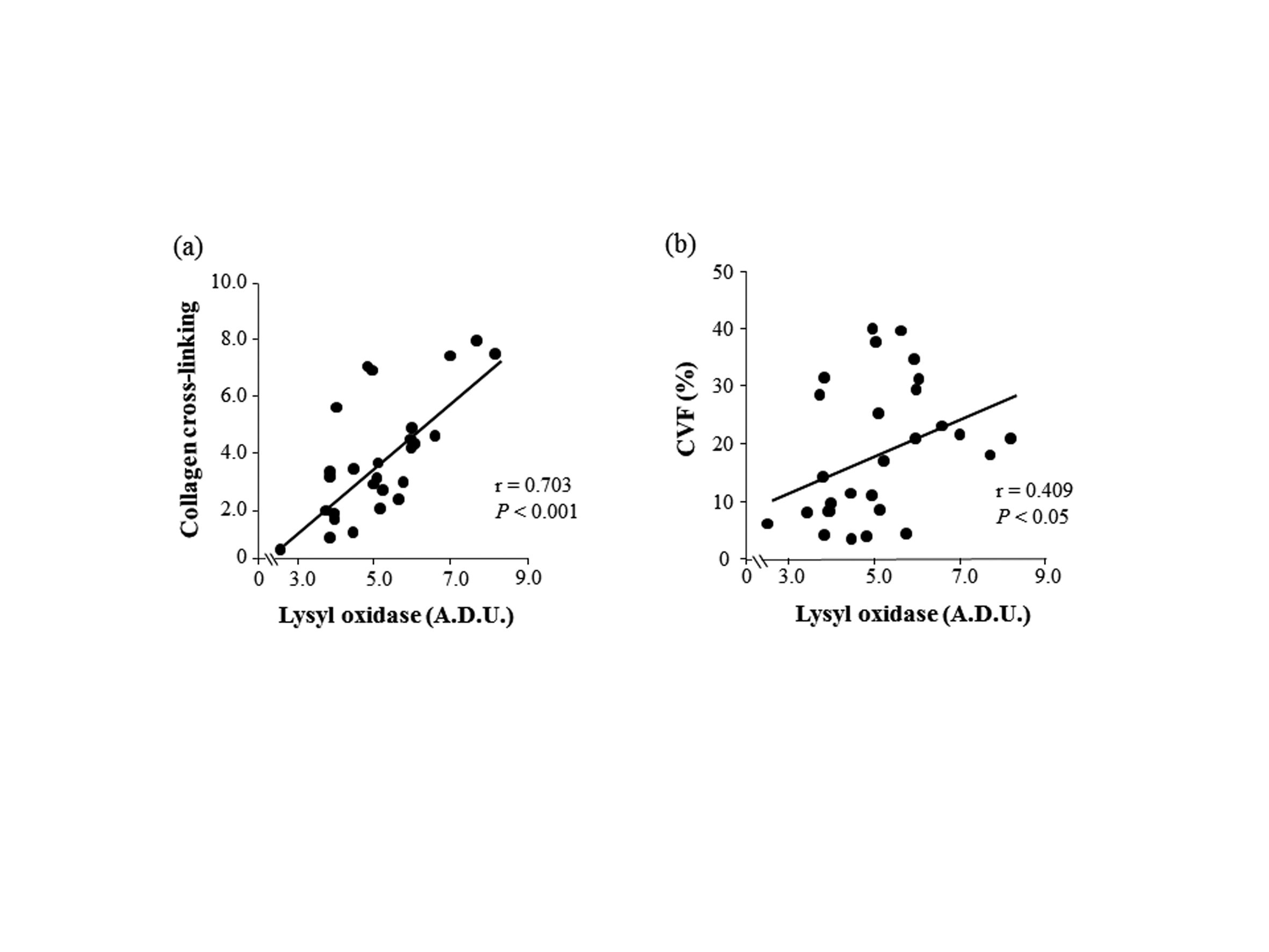
**

**Supplementary figure S1. Myocardial LOX protein is directly correlated with collagen cross-linking and collagen volume fraction in aortic stenosis patients.** Direct correlations of myocardial LOX protein with collagen cross-linking [linear fit: 1.141x – 2.241; panel (a)] and with collagen volume fraction (CVF) [linear fit: 3.209x + 1.647; panel (b)] in all patients with aortic stenosis. A.D.U. means arbitrary densitometric units. P values are for bivariate correlation analysis.


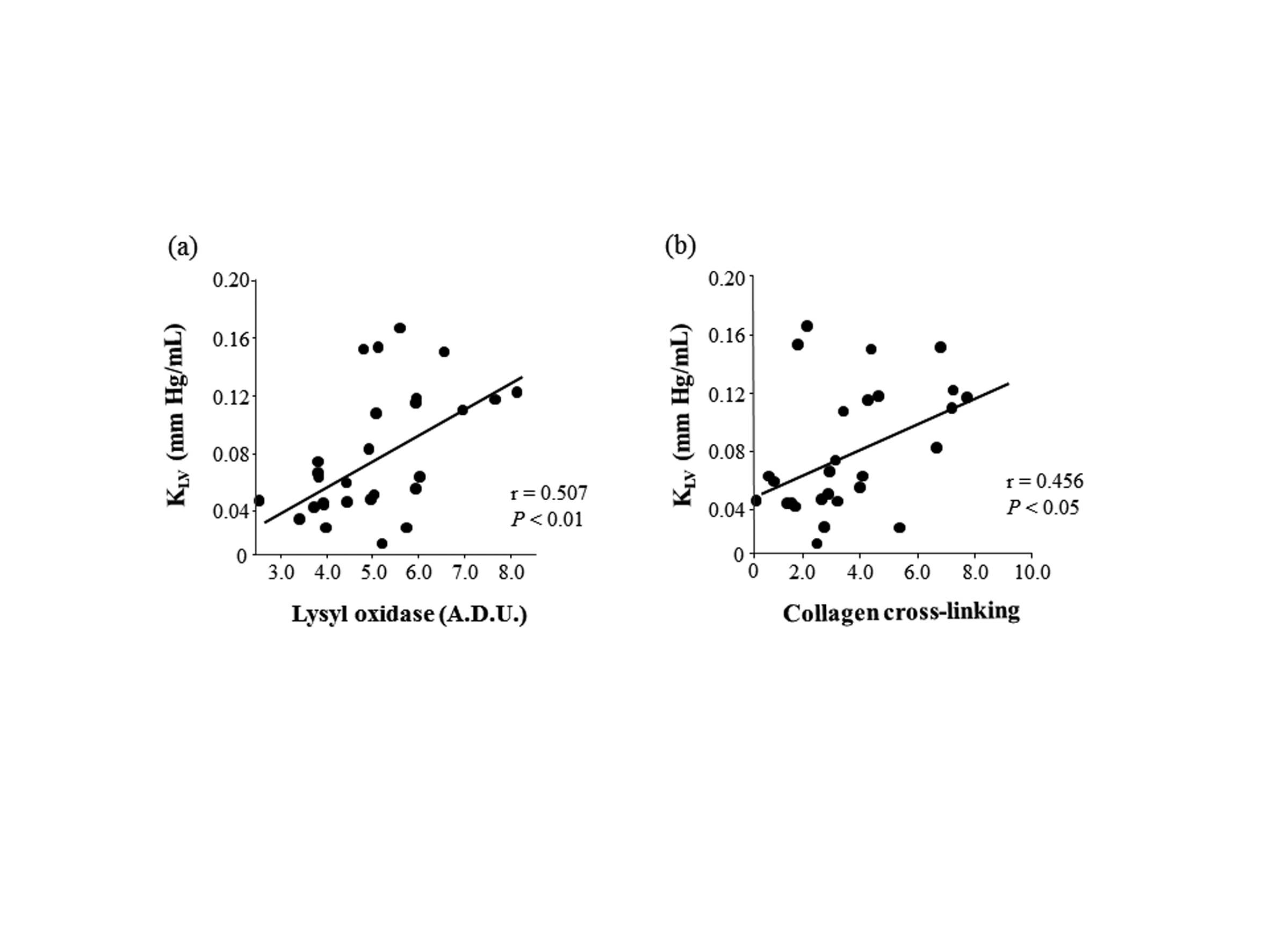


**Supplementary figure S2. Myocardial LOX protein and collagen cross-linking are directly correlated with left ventricular stiffness in aortic stenosis patients.** Direct correlations of K_LV_ with LOX [linear fit: 0.018x – 0.016; panel (a)] and with collagen cross-linking [linear fit: 0.009x + 0.046; panel (b)] in all patients with aortic stenosis. A.D.U. means arbitrary densitometric units. P values are for bivariate correlation analysis.
